# Comprehensive data on the mechanical properties and biodegradation profile of polylactide composites developed for hard tissue repairs

**DOI:** 10.1016/j.dib.2020.106107

**Published:** 2020-08-01

**Authors:** Abraham K. Aworinde, Samson O. Adeosun, Festus A. Oyawale, Eyere Emagbetere, Felix A. Ishola, Obafemi Olatunji, Stephen A. Akinlabi, Sunday O. Oyedepo, Oluseyi O. Ajayi, Esther T. Akinlabi

**Affiliations:** a Mechanical Engineering Department, College of Engineering, Covenant University, Ota, Nigeria; b Metallurgical and Materials Engineering Department, University of Lagos, Nigeria; c Mechanical Engineering Department, Federal University of Petroleum Resources, Effurun, Nigeria; d Mechanical Engineering Science Department, University of Johannesburg, South Africa; e Department of Mechanical Engineering, Walter Sisulu University, Butterworth Campus, Butterworth, South Africa

**Keywords:** Compressive properties, Hard tissue regeneration, Melt-blending technique, Predicted fracture toughness, Vickers microhardness

## Abstract

Polylactide (PLA), a biopolymer, was reinforced with three fillers (two organic reinforcements and one inorganic filler). The processing technique used to fabricate the composites was the melt-blending technique. The composites and the unreinforced PLA were subjected to microhardness, compression and biodegradation characterisations. Data obtained are presented in this article as raw data. Data from microhardness and compression tests were used to predict the fracture toughness. The biodegradation of the composites was also examined, and the data obtained reported in this article. The data presented in this article allow for a comprehensive understanding of the mechanical behaviour and the biodegradation profile of three composites of PLA with respect to their applications as biodegradable implants. It also helps in the selection of fillers for biopolymers such as PLA.

Specifications table

**Table:** 

Subject area	Material science
More specific subject area	Biomaterials
Type of data	Table
How data were acquired	Vickers microhardness tester, double column Instron universal testing machine with model number 3369 equipped with Bluehill software, and immersion in phosphate buffer solution
Data format	Raw Analysed
Parameter for data collection	Geometric progression, with the first term and the common ratio being 0.5 g and 2 respectively, was used to obtain the fillers’ weight percentages. Parameters (such as strength, modulus, fracture toughness, biodegradation, etc.) for the design of a biodegradable implant were considered in the characterisations.
Description of data collection	Melt-blending technique was used to develop three different PLA composites with chitin, chitosan and titanium (Ti-6Al-2Sn-2Mo-2Cr-0.25Si) powders as the reinforcements. Cylinders were produced from the composites and subjected to microhardness, compression and biodegradation tests.
Data source location	Centre for Energy Research and Development, Obafemi Awolowo University, Ile-Ife, Nigeria. University of Johannesburg, Auckland Park campus, South Africa. Covenant University, Ota, Nigeria.
Data accessibility	With the article
Related research article	Aworinde, A. K., Adeosun, S. O., Oyawale, F. A., Akinlabi, E. T., & Akinlabi, S. A. (2019). The Strength characteristics of Chitosan- and Titanium- Poly (L-lactic) Acid Based Composites. Journal of Physics: Conference Series, 1378, 022061. https://doi.org/10.1088/1742-6596/1378/2/022061

Value of the data

• These data are significant because they present the mechanical competencies and biodegradation profile of polylactide (PLA) reinforced with organic fillers (i.e., chitin and chitosan) and compared with PLA loaded with inorganic powder (i.e., Ti-6Al-2Sn-2Mo-2Cr-0.25Si) for applications in osteologic repairs
• Researchers, orthopaedists and institutions that are interested in the application of accelerated biodegradation can benefit from these data because the data help to understand the extent to which organic and inorganic fillers could influence the mechanical properties of PLA and its biodegradation tendencies
• These data can be reused for further insights and development of experiments by examining the influence of greater weight percentages of the fillers, organic ones in particular, on the biomechanical properties of PLA.
• The dataset can be applied in the short or long term because bone fracture and issues associated with the need for biodegradable materials are not one-off issues

## 1 Data description

The unreinforced polylactide, chitosan reinforced polylactide, chitin reinforced polylactide, and titanium reinforced polylactide have been abbreviated to PLA, PLA/Ch, PLA/Ct and PLA/Ti respectively for the purpose of terseness. The choice of chitin and chitosan, as reinforcements, is based on their hydrophobicity-reduction tendencies [1] and track record in the biomedical applications [2], [3], [4]. Ti-6Al-2Sn-2Mo-2Cr-0.25Si was considered as a filler because all its alloying elements are biocompatible. Besides, a similar alloy of titanium (i.e., Ti–6Al–2Sn–2Zr–2Mo–2Cr–0.25Si) has shown evidence of high corrosion resistance [5]. The mechanical properties of pure PLA and its composites are presented in Section 1.1, while Section 1.2 presents the analyses of variance (two-way ANOVA) of all the mechanical properties considered in this article. Section 1.3 is a record of the data got from the biodegradation test. The negative values in Sections 1.1 and 1.2 are indicative of the percentage decrease.

The Vickers microhardness values (H_V_), are presented in Table 1. Table 2 depicts half diameter of the indented section (*a*), and the largest lateral extension of cracks (C) observed during the microhardness test. These Tables comprehensively describe the microhardness properties of all the samples considered in this article as well as the measurements of parameters relating to the observed cracks during indentations. Table 1, for instance, gives the measure of the resistance of the samples to plastic deformation during indentation while Table 2 details the quantitative values of half of the diameter of the impression made by the indenter and the largest lateral extension (C) of the observed cracks. C was obtained by the addition of *a* to the measured length of microcracks [6] observed through the measuring microscope of the Vickers microhardness tester. These values (i.e., H_V_, *a* and C) allude to the ductility of PLA and its composites and are precursors to the fracture toughness of the samples.

**Table 1:** Vickers microhardness values.

Filler (wt%)	Hardness value (HV)			% increase in hardness of		
	PLA/Ch	PLA/Ct	PLA/Ti	PLA/Ct over PLA/Ch	PLA/Ti over PLA/Ct	PLA/Ti over PLA/Ch
0.00	68.6	68.6	68.6	0.0000	0.0000	0.0000
1.04	76.1	134	151.8	76.0841	13.2836	49.8683
2.08	90.2	135.5	161.8	50.2217	19.4096	44.2522
4.17	107.2	154.5	180.5	44.1231	16.8285	40.6094
8.33	148.4	213.1	268.1	43.5984	25.8095	44.6475
16.67	167.7	210.9	165.2	25.7603	-21.6690	-1.5133

**Table 2:** Half-diameter of the indented section, *a* (mm) and largest lateral extension of the crack, C (mm).

Filler (wt%)	a (mm)			C (mm)		
	PLA/Ch	PLA/Ct	PLA/Ti	PLA/Ch	PLA/Ct	PLA/Ti
0.00	25.7750	25.7750	25.7750	53.0917	53.0917	53.0917
1.04	20.7667	17.5917	17.0083	59.9333	58.3250	59.8917
2.08	21.8417	19.0000	15.5667	60.5250	57.0167	56.7833
4.17	20.7167	17.9333	18.7917	73.0500	62.9500	63.5083
8.33	18.0000	15.0750	16.9083	61.9833	59.1250	59.9750
16.67	16.0750	17.0583	18.2250	64.0250	55.4917	62.2750

Tables 3 and 4 are data obtained from the compression test. Table 3 shows the ultimate compressive strength of the samples. The values were obtained at the maximum compressive load and are necessary values in the determination of the ability of the samples to withstand compressive loads. Compressive moduli are presented in Table 4. The values were the slope of the stress-strain curves within the elastic region. These values summarised the stress-strain behaviour of PLA and its developed composites. The modulus of any material for hard tissues repairs, e.g., bone internal fixations, can help to understand if there would be a modulus mismatch. Table 5 collates the predicted fracture toughness values using Eqs. (2) and (3). The fracture toughness values obtained using Eq. (2) only used data from Vickers microhardness test, whereas Eq. (3) used data obtained from both microhardness and compression tests. These values reveal the quantitative ability of the samples to resist fracture in the presence of cracks.

**Table 3:** Ultimate compressive strength (UCS).

Filler (wt%)	UCS (MPa)			% increase in UCS of		
	PLA/Ch	PLA/Ct	PLA/Ti	PLA/Ct over PLA/Ch	PLA/Ti over PLA/Ct	PLA/Ti over PLA/Ch
0.00	24.75	24.75	24.75	0.0000	0.0000	0.0000
1.04	12.53	23.46	22.84	87.2565	−2.6450	45.1464
2.08	6.91	9.32	18.52	34.9998	98.6323	62.7079
4.17	13.82	4.89	13.44	−64.6299	174.8765	−2.8552
8.33	21.93	21.14	23.94	−3.6250	13.2707	8.3952
16.67	28.83	6.44	16.66	−77.6749	158.7551	−73.1084

**Table 4:** Compressive modulus (Ec).

Filler (wt%)	E_c_ (MPa)			% increase in E_c_ of		
	PLA/Ch	PLA/Ct	PLA/Ti	PLA/Ct over PLA/Ch	PLA/Ti over PLA/Ct	PLA/Ti over PLA/Ch
0.00	522.18	522.18	522.18	0.0000	0.0000	0.0000
1.04	376.34	444.61	546.13	18.1400	22.8342	31.0898
2.08	357.24	400.90	424.42	12.2212	5.8685	15.8298
4.17	419.96	513.47	416.79	22.2680	−18.8294	−0.7601
8.33	536.29	709.73	637.59	32.3418	−10.1646	15.8884
16.67	841.94	812.21	863.77	−3.5312	6.3484	2.5275

**Table 5:** Predicted fracture toughness.

Filler (wt. %)	Fracture toughness from Eq. (2)			Fracture toughness from Eq. (3)		
	PLA/Ch	PLA/Ct	PLA/Ti	PLA/Ch	PLA/Ct	PLA/Ti
0.00	0.1840	0.1840	0.1840	0.0410	0.0410	0.0410
1.04	0.1534	0.1598	0.1536	0.0352	0.0402	0.0434
2.08	0.1512	0.1654	0.1663	0.0345	0.0383	0.0409
4.17	0.1140	0.1425	0.1407	0.0340	0.0405	0.0366
8.33	0.1459	0.1566	0.1533	0.0416	0.0498	0.0447
16.67	0.1390	0.1722	0.1449	0.0503	0.0519	0.0502

### 1.1 Mechanical properties

Table 1, Table 2, Table 3, Table 4, Table 5.

### 1.2 Analysis of variance on the mechanical properties

Table 6, Table 7, Table 8, Table 9, Table 10 are the analyses (using two-way ANOVA) of the data on the Vickers hardness, ultimate compressive strength, compressive modulus, fracture toughness values obtained from Eq. (2), and fracture toughness values obtained using Eq. (3) respectively. The summary of the analyses of variance on the mechanical properties of the samples is in Table 12. These analyses relate two factors (i.e., the variation in the weight per cent of the fillers and differences in the structural strengths of the fillers) to the mechanical properties of PLA and its composites.

**Table 6:** Analysis of variance on the hardness properties of composites.

Summary	Count	Sum	Average	Variance		
0	3	205.8	68.6	0		
1.04	3	361.9	120.6333	1566.623		
2.08	3	387.5	129.1667	1311.723		
4.17	3	442.2	147.4	1381.03		
8.33	3	629.6	209.8667	3589.863		
16.67	3	543.8	181.2667	660.1633		
PLA/Ch	6	658.2	109.7	1613.272		
PLA/Ct	6	916.6	152.7667	2951.391		
PLA/Ti	6	996	166	4068.268		

ANOVA						
Source of Variation	SS	df	MS	F	P-value	F crit
Rows	36544.94	5	7308.989	11.04125	0.000811	3.325835
Columns	10399.1	2	5199.549	7.854649	0.008903	4.102821
Error	6619.709	10	661.9709			
Total	53563.75	17

**Table 7:** Analysis of variance on the compressive strength of composites.

Summary	Count	Sum	Average	Variance		
0	3	74.25	24.75	0		
1.04	3	58.83	19.61	37.6909		
2.08	3	34.75	11.58333	37.54003		
4.17	3	32.15	10.71667	25.49863		
8.33	3	67.01	22.33667	2.084033		
16.67	3	51.93	17.31	125.6449		
PLA/Ch	6	108.77	18.12833	69.71578		
PLA/Ct	6	90	15	82.41636		
PLA/Ti	6	120.15	20.025	20.50551		

ANOVA						
Source of Variation	SS	df	MS	F	*P*-value	F crit
Rows	483.5401	5	96.70802	2.547307	0.097684	3.325835
Columns	77.26888	2	38.63444	1.017638	0.396022	4.102821
Error	379.6481	10	37.96481			
Total	940.4571	17

**Table 8:** Analysis of variance on the compressive modulus of composites.

Summary	Count	Sum	Average	Variance		
0	3	1566.526	522.1754	0		
1.04	3	1367.086	455.6952	7299.444		
2.08	3	1182.562	394.1872	1162.256		
4.17	3	1350.219	450.0731	3017.178		
8.33	3	1883.609	627.8698	7591.635		
16.67	3	2517.926	839.3085	669.8638		
PLA/Ch	6	3053.942	508.9903	32065.26		
PLA/Ct	6	3403.1	567.1833	25588.82		
PLA/Ti	6	3410.886	568.481	27675.96		

ANOVA						
Source of Variation	SS	df	MS	F	*P*-value	F crit
Rows	401024	5	80204.79	31.29792	8.49E−06	3.325835
Columns	13854.51	2	6927.255	2.703188	0.115212	4.102821
Error	25626.24	10	2562.624			
Total	440504.7	17

**Table 9:** Analysis of variance on the energy at maximum strength of composites.

Summary	Count	Sum	Average	Variance		
0.00	3	4.71	1.5700	0.0000		
1.04	3	1.68	0.5600	0.1101		
2.08	3	0.84	0.2800	0.0553		
4.17	3	1.24	0.4133	0.1332		
8.33	3	2.69	0.8967	0.1204		
16.67	3	1.77	0.5900	0.2457		
PLA/Ch	6	4.20	0.7000	0.3489		
PLA/Ct	6	3.13	0.5217	0.3073		
PLA/Ti	6	5.60	0.9333	0.1577		

ANOVA						
Source of Variation	SS	df	MS	F	*P*-value	F crit
Rows	3.2515	5	0.6503	7.9489	0.0029	3.3258
Columns	0.5114	2	0.2557	3.1257	0.0882	4.1028
Error	0.8181	10	0.0818			
Total	4.5811	17

**Table 10:** Analysis of variance on the fracture toughness values of composites obtained using Eq. (2).

Summary	Count	Sum	Average	Variance		
0	3	0.552485	0.184162	0		
1.04	3	0.46719	0.15573	1.33E−05		
2.08	3	0.483273	0.161091	7.22E−05		
4.17	3	0.397511	0.132504	0.000255		
8.33	3	0.456081	0.152027	3.01E−05		
16.67	3	0.456375	0.152125	0.000315		
PLA/Ch (Eq 2)	6	0.888166	0.148028	0.000516		
PLA/Ct (Eq 2)	6	0.981267	0.163545	0.000201		
PLA/Ti (Eq 2)	6	0.94348	0.157247	0.000252		

ANOVA						
Source of Variation	SS	df	MS	F	*P*-value	F crit
Rows	0.004204	5	0.000841	13.13114	0.000397	3.325835
Columns	0.000731	2	0.000365	5.706376	0.022215	4.102821
Error	0.00064	10	6.4E-05			
Total	0.005576	17

**Table 11:** Analysis of variance on the fracture toughness values of composites obtained using Eq. (3).

Summary	Count	Sum	Average	Variance		
0	3	0.123044	0.041015	0		
1.04	3	0.11876	0.039587	1.73E−05		
2.08	3	0.11367	0.03789	1.02E−05		
4.17	3	0.111018	0.037006	1.07E−05		
8.33	3	0.136021	0.04534	1.69E−05		
16.67	3	0.152404	0.050801	9.66E−07		
PLA/Ch	6	0.236597	0.039433	3.95E−05		
PLA/Ct	6	0.261624	0.043604	3.28E−05		
PLA/Ti	6	0.256696	0.042783	2.07E−05		

ANOVA						
Source of Variation	SS	df	MS	F	*P*-value	F crit
Rows	0.000412	5	8.23E-05	15.38525	0.000203	3.325835
Columns	5.86E−05	2	2.93E-05	5.47389	0.024792	4.102821
Error	5.35E−05	10	5.35E-06			
Total	0.000524	17

**Table 12:** Summary of the analysis of variance on the mechanical properties of the developed composites.

Mechanical property	Effect of fillers’ weight per cent			Effect of fillers’ structural strengths		
	*F*	*P-value*	*F crit*	*F*	*P-value*	*F crit*
Hardness	11.04	$8.11\;\times 10^{-4}$	3.33	7.85	$8.90\;\times 10^{-3}$	4.10
Compressive strength	2.55	$9.77\;\times 10^{-2}$	3.33	1.02	$3.96\;\times 10^{-1}$	4.10
Compressive modulus	31.30	$8.49\;\times 10^{-6}$	3.33	2.70	$1.15\;\times 10^{-1}$	4.10
Fracture toughness (Eq. 2)	13.13	$3.97\;\times 10^{-4}$	3.33	5.71	$2.22\;\times 10^{-2}$	4.10
Fracture toughness (Eq. 3)	15.39	$2.03\;\times 10^{-4}$	3.33	5.47	$2.48\;\times 10^{-2}$	4.10

### 1.3 Biodegradation Profile of the composites

In Table 13, the changes in mass observed during the biodegradation test are recorded. The initial masses of the samples, masses after four weeks and eventual masses after ten weeks of immersion of the samples in the phosphate buffer solution are all contained in Table 13. The masses gained or lost after four and ten weeks of immersion are shown in Table 14 in percentage terms. These data describe the onset of biodegradation by hydrolytic degradation [7].

**Table 13:** Mass change during biodegradation.

Filler (wt%)	Initial mass (g)			Mass (g) after 4 weeks			Mass (g) after 10 weeks		
	PLA/Ch	PLA/Ct	PLA/Ti	PLA/Ch	PLA/Ct	PLA/Ti	PLA/Ch	PLA/Ct	PLA/Ti
0.00	1.2473	1.2473	1.2473	1.3935	1.3935	1.3935	1.3251	1.3251	1.3251
1.04	1.2094	1.3011	1.1973	1.3607	1.4064	1.2164	1.2928	1.4163	1.2776
2.08	1.0173	1.3290	1.1813	1.1878	1.4636	1.2716	1.1709	1.4110	1.3653
4.17	1.1857	1.1184	1.2092	1.2473	1.2592	1.2991	1.2585	1.2248	1.3696
8.33	1.3278	1.3618	1.1859	1.4026	1.4878	1.2553	1.4511	1.4790	1.3485
16.67	1.2272	1.4951	1.3791	1.3857	1.6863	1.4607	1.3722	1.8330	1.5073

**Table 14:** Percentage changes in mass after immersion in Phosphate Buffer Solution (PBS).

Filler (wt%)	Mass (g) after 4 weeks			Mass (g) after 10 weeks		
	PLA/Ch	PLA/Ct	PLA/Ti	PLA/Ch	PLA/Ct	PLA/Ti
0.00	11.7213	11.7213	11.7213	6.2375	6.2375	6.2375
1.04	12.5103	8.0932	1.5953	6.8960	8.8540	6.7068
2.08	16.7600	10.1279	7.6441	15.0988	6.1700	15.5761
4.17	5.1952	12.5894	7.4347	6.1398	9.5136	13.2650
8.33	5.6334	9.2525	5.8521	9.2860	8.6063	13.7111
16.67	12.9156	12.7884	5.9169	11.8155	22.6005	9.2959

## 2 Experimental design, materials and methods

Polylactide (PLA) with the molecular weight of 144 g/mol and the overall lactide purity ≥99.5% was purchased from NatureWorks, China. Chitin and chitosan were obtained via chemical extraction processes from shrimp shells while titanium powder (Ti–6Al–2Sn–2Mo–2Cr–0.25Si) was purchased from TLS Technik GmbH & Co. Spezialpulver KG, Bitterfeld-Wolfen, Germany. While PLA served as the matrix, chitin, chitosan and titanium powder served as the reinforcements. The matrix was melt-blended with each of the fillers at the weight percentages shown in Table 15. Although there are several polymer composites processing technique [8], [9], [10], [11], the melt-blending technique was used because it is environmentally benign, cost-effective, best for mass production, toxin-free [12] and allows for the addition of higher weight per cent of fillers. The weight per cent formulation of the fillers was obtained from the mass of the fillers (Table 15) according to Eq. (1). The stirring speed of 60 rpm was used to ensure a fairly homogenous mix of the fillers with the matrix. Each of the molten composites was mould-pressed at the pouring temperature of 290 °C to form solid cylinders with 12.5 mm diameter and 7.0 mm length.

(1) $$M_{n}=ar^{n-1}$$

where:

*M_n_* = mass of the nth term
*a* = the starting mass of the filler (i.e., 0.5 g)
*r* = common ratio (i.e., 2)
*n* = nth term of mass of the filler

**Table 15:** The formulation of the fillers’ weight per cent.

n	PLA (g)	PLA (wt. %)	Filler (g)	Filler (wt%)
0	88.0	100.00	0.0	0.00
1	87.5	98.96	0.5	1.04
2	87.0	97.92	1.0	2.08
3	86.0	95.83	2.0	4.17
4	84.0	91.67	4.0	8.33
5	80.0	83.33	8.0	16.67

### 2.1 Mechanical characterisation

The unreinforced and developed solid composites were subjected to Vickers microhardness test with indentation load of 100 kgf for 10 s dwell time (except for PLA/Ti at 16.67 wt% which took 15 s). The microhardness machine used was located at the Mechanical Engineering Science Department, University of Johannesburg, Auckland Park Campus, South Africa. The Vickers hardness values and lateral extended micro-cracks were measured and recorded. The compression test was done using a double column Instron universal testing machine with model number 3369 (equipped with Bluehill software for data acquisition) located at Centre for Energy Research and Development (CERD) at Obafemi Awolowo University, Ile-Ife, in Nigeria. Fracture toughness was predicted from the data obtained from compression and Vickers microhardness tests using Eqs. (2) and (3) [6].

(2) $$K_{\text{IC}}=0.0726\frac{P}{C^{\frac{3}{2}}}$$

(3) $$K_{\text{IC}}=0.0089{\left(\frac{E}{H_{V}}\right)}^{\frac{2}{5}}\left(\frac{P}{aC^{\frac{1}{2}}}\right)$$

where

K_IC_ = fracture toughness (MPa.m^0.5^)
P = indentation loading (N)
E = Young's modulus (GPa)
H_V_ = Vickers hardness (GPa)
*a* = half-diameter of the indented section (mm)
C = largest lateral extension of the crack (mm)

### 2.2 Biodegradation Test

20 ml of Phosphate Buffer Solution (PBS) with 7.4 pH was measured into each test tube and kept in an oven with a preset temperature of 36.5 °C. The test tubes were left in the oven for about 30 min to ensure the conditioning of the PBS to 36.5 °C [13]. The weighed samples were then immersed in 20 ml of PBS. The test tubes were returned into the oven, and the temperature maintained at 36.5 °C.

The biodegradation test was left on for ten (10) weeks. Changes in mass, which is considered as the progress of biodegradation [7], were measured after the first four (4) weeks and at the end of the tenth week. The percentage change in mass after weeks of immersion, *M_Δ_*, was calculated for every sample using Eq. (4).

(4) $$M_{\Delta }\;=\;\frac{M_{f}\;-\;M_{i}}{M_{i}}\;\times \;100\;\%$$

where

$M_{\Delta }=\text{percentage}\;\text{change}\;\text{in}\;\text{mass}$
$M_{i}=\text{initial}\;\text{mass}\;\text{before}\;\text{immersion}$
$M_{f}=\text{final}\;\text{mass}\;\text{after}\;\text{immersion}$

## Declaration of Competing Interest

The authors declare that they have no known competing financial interests or personal relationships that could have appeared to influence the work reported in this paper.

## Appendix Supplementary materials

Image, application 1
